# The Vulnerability of People to Damaging Hydrogeological Events in the Calabria Region (Southern Italy)

**DOI:** 10.3390/ijerph15010048

**Published:** 2017-12-29

**Authors:** Olga Petrucci, Paola Salvati, Luigi Aceto, Cinzia Bianchi, Angela Aurora Pasqua, Mauro Rossi, Fausto Guzzetti

**Affiliations:** 1 CNR IRPI (Consiglio Nazionale delle Ricerche, Istituto di Ricerca per la Protezione Idrogeologica), via Cavour 4/6, I-87036 Rende (CS), Italy; aceto@irpi.cnr.it (L.A.); aurora.angela.pasqua@irpi.cnr.it (A.A.P.); 2 CNR IRPI (Consiglio Nazionale delle Ricerche, Istituto di Ricerca per la Protezione Idrogeologica), via Madonna Alta 126, I-06128 Perugia, Italy; paola.salvati@irpi.cnr.it (P.S.); cinzia.bianchi@irpi.cnr.it (C.B.); mauro.rossi@irpi.cnr.it (M.R.); Fausto.Guzzetti@irpi.cnr.it (F.G.)

**Keywords:** floods, landslides, victims

## Abstract

*Background:* Damaging Hydrogeological Events (DHEs) are severe weather periods during which floods, landslides, lightning, windstorms, hail or storm surges can harm people. Climate change is expected to increase the frequency/intensity of DHEs and, consequently, the potential harm to people. *Method:* We investigated the impacts of DHEs on people in Calabria (Italy) over 37 years (1980–2016). Data on 7288 people physically affected by DHEs were gathered from the systematic analysis of regional newspapers and collected in the database named PEOPLE. The damage was codified in three severity levels as follows: fatalities (people who were killed), injured (people who suffered physical harm) and involved (people who were present at the place where an accident occurred but survived and were not harmed). During the study period, we recorded 68 fatalities, 566 injured and 6654 people involved in the events. *Results:* Males were more frequently killed, injured and involved than females, and females who suffered fatalities were older than males who suffered fatalities, perhaps indicating that younger females tended to be more cautious than same-aged males, while older females showed an intrinsic greater vulnerability. Involved people were younger than injured people and fatalities, suggesting that younger people show greater promptness in reacting to dangerous situations. Floods caused the majority of the fatalities, injured and involved people, followed by landslides. Lightning was the most dangerous phenomenon, and it affected a relatively low number of people, killing 11.63% of them and causing injuries to 37.2%. Fatalities and injuries mainly occurred outdoors, largely along roads. In contrast, people indoors, essentially in public or private buildings, were more frequently involved without suffering harm. Being “dragged by water/mud” and “surrounded by water/mud”, respectively, represented the two extremes of dynamic dangerousness. The dragging effect of rapid-flowing water totally or partially obstructed the attempts of people to save their lives. In contrast, people surrounded by steady water/mud encountered difficulties but ultimately could survive. *Conclusions:* The study outcomes can be used in informational campaigns to increase risk awareness among both administrators and citizens and to improve community resilience, particularly in promoting self-protective behaviors and avoiding the underestimation of hazardous situations.

## 1. Introduction

Damaging Hydrogeological Events (DHEs) are episodes of severe weather during which landslides, floods [1] and other potentially harmful phenomena, such as lightning, windstorms, hail and storm surges can cause physical damage to people [2,3].

Between 1980 and 2009, floods killed more than 539,811 and injured more than 361,974 people globally [4]. Climate change may cause a further increase in flood hazard probability and magnitude, while demographic and economic development will cause a continuous increase in the vulnerability of settlements and infrastructure located on floodplains and in coastal areas [5]. Between 2007 and 2012, landslides killed 4700 people in 112 countries, with a peak in landslide fatalities in August in the Northern Hemisphere attributed to the Asian monsoon and cyclone seasons [6]. Disastrous flood and landslide impacts can be reduced if people are informed and persuaded towards a culture of disaster prevention and resilience; this education would require the collection, compilation and dissemination of relevant knowledge and information on hazards and vulnerabilities [5]. Detailed and organized information on geographical and temporal distribution of DHEs and of their consequences is a foundation needed to implement national communication strategies and preparedness programs. Improving peoples’ behavior, teaching the avoidance of risky places and actions, and promoting safer conduct can be effective preparedness measures that help minimize (if not eliminate) possible harm during future DHEs.

For a period of 37 years, data on DHEs causing damage to people in the Calabria Region (Southern Italy) were collected and systematized in PEOPLE, a database that we specifically designed to collect data mined from newspapers and concerning damage to people caused by DHEs. The damage was classified into three severity levels as follows: “fatalities” (people who were killed), “injured” (people who suffered physical harm), and “involved” (people present at the place where the accidents occurred but who survived and were not injured).

In this paper, we analyze the information stored in PEOPLE to identify different types of interactions, deadly or not, between people and events, to obtain valuable elements that can be used in informational campaigns to improve human behavior and reduce fatalities and injuries.

The significance of the PEOPLE database and this corresponding paper is in following points:

- The innovative approach, based on the classification of damage to people into three severity levels, and their comparative analysis aiming to identify the phenomena and circumstances causing either the maximum damage (fatalities) or no damage (involved people). In fact, literature largely only focuses on fatalities; however, the analysis of data on involved people is an innovative element introduced in Aceto et al. [7] and is improved upon in this paper.
- The identification of victim profiles in terms of age, gender, and severity of suffered damage.
- The analysis of people-phenomena interaction in the regional circumstances, in terms of damaging phenomena and peoples’ behavior during DHEs, with an overview of more dangerous and/or frequent circumstances in which people were affected.
- The repeatability of the methodological approach in other regions.
- The public availability of the database PEOPLE (The Database PEOPLE 2 is available on Mendely, http://dx.doi.org/10.17632/99knpdb6yp.1.), representing a resource to other scientists working on the same topic in other geographical frameworks.

The paper starts by reviewing literature on the fatalities caused by floods, landslides, and secondary phenomena (in terms of damage) acting during a DHE. Then, after the introduction of the study area, the Materials and Methods Section presents the data gathering and the structure of PEOPLE database. In the following sections, the data are presented and discussed, and the main conclusions are finally summarized.

## 2. Literature on Flood and Landslide Fatalities

Scientific literature classifies the effects of natural hazards on people as short- or long-term impacts [8]. Dangerous features of landslides and floods are different but can occur simultaneously during a DHE, with sequential or cascading effects that create multi-risk conditions to people, especially if they are outdoors and moving. The great majority of papers focus on flood victims, while the analysis of a landslide’s effects on people is less frequent, and, generally, it is a complementary element of the articles and does not represent the main focus. Literature usually analyzes only fatalities of people who lost their life due to either floods or landslides, while people injured are rarely reported, and involved people are never considered. In the following sections, we review the scientific literature on landslide and flood fatalities.

### 2.1. Flood Fatalities

Based on data from the decade 2000–2010, deaths caused by floods in low- versus high-income countries show a ratio of 23:1 [9]. There are opposite trends for developed/high-income and undeveloped/low-income countries. Despite the fact that, in lower-income countries, the adaptation of “learning from the past” seems stronger, the average death tolls are still higher than those in higher-income countries [10,11]. In developed countries, moderate decreases in flood fatality were reported in Switzerland [12], Australia [13] and Greece [14]. In developed communities, males experience a higher flood mortality than that of females, as detected by Jonkmann and Kelman [15] in Europe and the USA, while females suffered higher mortality in low-income countries [16].

In Table 1, we list the findings of recent studies on flood fatalities, excluding the following types of research: (a) studies on single flood events, because these do not analyze long observation periods as our research does; (b) studies on motor-related drowning, because the present paper analyzes “all” fatalities caused by floods, in addition to the subsample related to car accidents; and (c) papers on the long-term effects on human health (mortality as a result of malnutrition, diseases, effects of mental disorders or illnesses from flood-induced contamination of water supplies) (e.g., [17]). We analyzed 13 papers published between 1999 and 2018. The longest study periods concern Australia [18], followed by Portugal [19] and Switzerland [12]. The papers present the total number of fatalities that occurred during the study periods in large territories, such as a continent or a small region, and almost all of the supplied information on the percentage of male and female victims. Generally, males were consistently more affected than females, except between 1965 and 2014 in Italy, where this difference was not as large (males were 57.6% and females 42.4% of flood fatalities) [20]. In contrast, details regarding the circumstances in which people died are not frequently described, except for papers addressing motorists killed by floods, which is the most developed sector of literature regarding flood fatalities.

### 2.2. Landslide Fatalities

The effects of landslides are spatially circumscribed, and, maybe for this reason, the loss of life is globally poorly quantified and analyzed. Whereas large-scale hazards such as floods or hurricanes have global catalogues of frequency and losses, few inventories have been developed for relatively smaller-scale hazards such as landslides. Nevertheless, these phenomena have an impact on people, especially at certain latitudes, particularly in urbanistic frameworks. According to Petley [27], between 2004 and 2010, 2620 fatal landslides were recorded worldwide, causing 32,322 fatalities, mainly in Asia and China, with a peak during the summer months in the Northern Hemisphere.

Inventories of landslide fatalities are infrequent; data on landslide victims are included in landslide inventories devoted to purposes other than the assessment of human impact. Generally, the number of fatalities is used as a measure of landslide impact, thus it is simply quoted without supplying either the gender or age of victims nor their death circumstances, as in the case of the Global Landslide Catalogue [6]. Table 2 summarizes studies based on landslide fatality inventories that cover a period sufficiently wide to be compared to those analyzed in this paper. The most frequently surveyed element is the number of landslides causing fatalities, which is reported in almost all the analyzed papers. In contrast, among the listed papers, only three papers reported data regarding the gender of fatalities [12,19,20]; in all cases, male fatalities were more numerous than female fatalities.

### 2.3. Secondary Phenomena

Most natural disasters are triggered by multi-hazards that occur simultaneously or sequentially rather than singly (e.g., landslides and floods), thus resulting in more severe consequences. The multi-hazard approach is not as common in the literature. It can be found in studies concerning climate change and the possible exacerbation of climate extremes (wind, storms, storm surges and floods) [34]. Moreover, multi-hazards often characterize operative systems managing the real world of disasters, even if the hazards are not considered to be acting simultaneously. One example is FEMA’s Hazus-MH, a nationally applicable methodology that contains models for estimating potential losses from earthquakes, floods, and hurricanes. Hazus-MH uses Geographic Information Systems that allow users to visualize the spatial relationships between populations and fixed geographic assets for the specific hazard being modeled, which is a crucial function in pre-disaster planning (https://www.fema.gov/hazus).

The literature often addresses wind damage, with a focus on economic damage [35] in European countries or in the USA [36], without any detail of the harm to people. Other studies analyze the temporal evolution of fatalities related to more complex phenomena, i.e., tropical cyclones that occurred in China between 1951 and 2008, even if details regarding the way in which people died is not provided [37]. Similarly, Moore and Dixon [38] reported 306 injuries and 22 fatalities from 1995 to 2009 caused by tropical cyclone-tornados in the United States, without reporting the ultimate causes of death/injury. Likewise, systematic inventories of lightning-related deaths are null or not sufficient in many regions of the world [39], and, consequently, studies on fatalities and injuries caused by lightning are infrequent. Zhang et al. [40] reported 5033 deaths and 4670 injuries caused by lightning in China from 1997 to 2009. They found a typical seasonal distribution of victims and highlighted farmland as the most frequent casualty location. Other studies have focused on geographic regions characterized by higher rates of lightning-related mortality, such as Northern Malawi [41].

## 3. The Study Area

**Figure 1:**
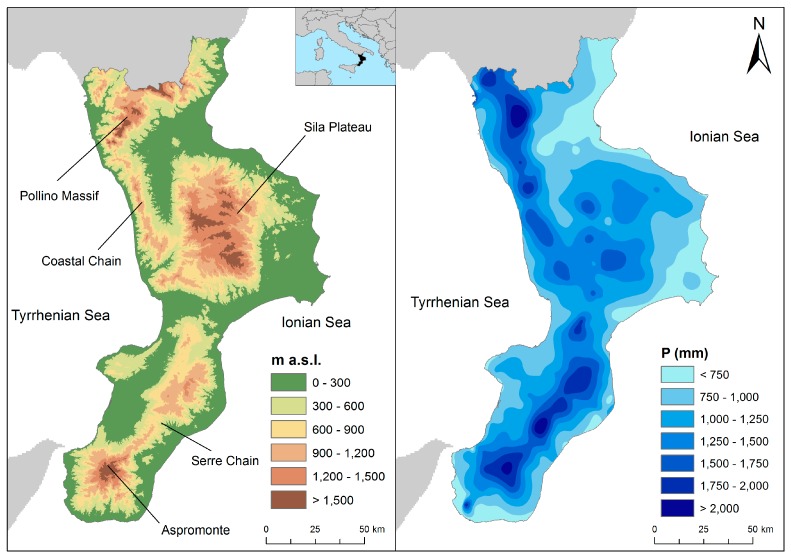
Digital elevation model (**left**); and annual rainfall (**right**) of Calabria region (Italy).

Our study area is Calabria, the southernmost region in Italy, with an area of 15,080 km^2^. According to the National Institute of Statistics (ISTAT, Chicago, IL, USA, http://www.istat.it/it/), the region has 1,970,521 inhabitants, 49% males and 51% females, living in 409 municipalities. Quaternary tectonic uplift, which is still active, shapes the regional morphology, the elevation of which ranges from sea level to 2260 m. The region is made of allochthonous crystalline rocks, Palaeozoic to Jurassic in age, stacked over carbonate units during the Middle Miocene, with Neogene flysch filling tectonic depressions [42]. The annual rainfall (average: 1150 mm) depends on elevation, with the mountainous sectors being wetter (>2000 mm) than the coastal areas (<500 mm) (Figure 1). From October to March, approximately 70% of the rainfalls often clustered in series of close cloudbursts that in a few days can reach more than 50% of the average annual rain. These severe rainy periods often originate DHEs, which cause tremendous damage to both properties and people [43]. Official loss-data collection concerning either economic damage or effects on people are not available.

## 4. Materials and Methods

As typically performed in geomorphologic research with a focus on the occurrence and impacts of geomorphologic hazards [44], in this research, data are gathered from documentary sources. The study is based on the systematic collection of data on damage caused by DHEs to people in a selected region over a long study period. The analysis of data does not aim to test an existing hypothesis but rather to collect and explain all the information that can be gathered from the sampling of the data collected. This qualitative research approach can be assimilated to the Grounded Theory Approach, a method of research accepted throughout the social sciences and nursing, which is described as “the discovery of emerging patterns in data, with the aim of seeking to generate theory from the research situation in the field, as it is” [45]. From the narrative of the accidents, we selected information that was disaggregated and systematized in the fields of PEOPLE, the database specifically developed to collect data mined from newspapers and concerning damage to people caused by DHEs. Data regarding cases occurring between 2000 and 2015 were obtained from a published section of PEOPLE [7]. For the period 1980–1999, we completed the database by the systematic survey of approximately 7300 daily editions of a regional newspaper named *La Gazzetta del Sud*, the only newspaper published continuously during that period.

### 4.1. PEOPLE Database

PEOPLE was designed to collect and organize data on the interactions between DHEs and the population. The database includes different fields grouped into sections described as follows:

#### 4.1.1. The “Event Identification”

This section contains the following fields:

(a) Time of the event: This contains the year (YYYY), month (MM) and day (DD) in which the damaging event occurred, and the identification number of each record, ID, composed of the sequence of year (YYYY), month (MM), and day (DD) of the event and the progressive record number (##) (Table 3).
(b) Type of phenomenon: In Calabria, the phenomena that harmed people during the study period are as follows: flood, urban flooding, landslide, rock fall, road collapse, windstorm, lightning, storm surge, and others (hail and thunderstorm). *Urban flooding* corresponds to *pluvial or rainfall floods* [5] that occur when heavy rainfall creates a flood event independent of an overflowing water body. This typically occurs in urban environments when the local drainage system is not capable of collecting and conveying surface runoff. The phenomenon classified as *road collapse* is the unexpected sinking of road caused by landslides, stream erosion or piping.
(c) Victim identification: This reports the name, surname, gender and age of the victims. Name and surname, when available, allow the exact identification of the people affected, avoiding double counts that can occur in cases where only generic descriptions were available (e.g., “a man died”).
(d) Damage severity: According to the severity of damage, persons are classified as follows: fatalities, injured, and involved people. The latter are people that were present at the place of the accident but survived without suffering physical damage. They may be: (a) those who displayed hazardous behavior; (b) those who were able to protect themselves; or (c) people who acted as nonprofessional rescuers. Especially in the case of severe events, newspapers do not report the exact number of involved people and often use collective measures (e.g., “tens of people”). We translated the colloquial “frequency words” [46] into numbers to obtain a rough assessment of the amount of people involved. We assumed some/a few people ≈ 4; several people ≈ 8; numerous/tens of people ≈ 10; many people ≈ 15; more than one hundred people ≈ 100.

#### 4.1.2. The “Victim-Event Interaction”

This contains the following fields:

(a) Place where the damage occurred, sorted into five indoor and nine outdoor locations (Table 3).
(b) Condition of people at the moment they were affected, classified into 12 types.
(c) Activity carried out by victims at the moment of the event, summarized into 10 types.
(d) Dynamic of people-event interaction, sorted into 18 types.

#### 4.1.3. The “Effects on People”

This includes the following two fields:

(a) Causes of death, sorted into 8 types.
(b) Types of injuries, sorted into 15 types.

It must be taken into account that the categorizations introduced in PEOPLE were carried out using events that actually occurred in the study region during the analyzed period. Thus, in geographical frameworks where different situations occur, the list must be updated accordingly. For example, in some countries, people during floods can suffer snake bites [47], but this circumstance did not occur in the study region during the analyzed period.

## 5. Data Presentation

In Calabria, during the 37-year study period, 7288 people (#PEO) were in some way physically affected during 740 events (#EV): 68 (0.93%) died, 566 (7.77%) were injured, and 6654 (91.30%) were involved in the events without suffering physical damage. Fatalities occurred during 44 events, with 1.55 victims per event, on average. The injured occurred during 153 events, with, on average, 3.70 per event. The events with people involved numbered 631, with 10.55 people involved per event, on average (Table 4). It should be noted that, typically, each event can cause either one or more than one type of effect on people (e.g., fatalities and injured or injured and involved people).

Approximately 200 people per year (196.97) were in some way affected by DHEs, with the majority not suffering physical damage (179.84 involved). The ratio of “people involved/fatalities”, is equal to 95.03, corresponding to one fatality for every 95 people involved, on average. The ratio of “people involved/injured”, is equal to 11.75, corresponding to approximately one injured person for every 10 people involved. Thus, when rescaled to 100, during the study period, approximately 10 of every 100 people were injured and 1 fatality occurred.

The term “unknown cases” refers to cases of fatalities, injured or involved people for which a specific variable (e.g., age of fatality, or dynamic) is unknown. The percentage of unknown cases is used to classify the reliability of each variable (Table 4), allowing for the assessment of the robustness of findings for each variable. According to the percentage of unknown cases, the reliability is labeled as follows: very high: 0–5%; high: 5.1–20%; medium: 20.1–40%; low: 40.1–60%; very low: >60%.

The completeness of data generally depends on the severity of damage. In fact, the most complete information concerns fatalities, for which the reliability of each variable is greater than that characterizing injured and involved people. Thus, data regarding injured and involved people is less reliable than that for fatalities. Nevertheless, the relative percentage of the three severity levels allows one to sort circumstances in terms of dangerousness, while details regarding behavior of the injured and involved people allow one to understand the way in which they managed to avoid death or injuries.

### 5.1. Temporal Trend

**Figure 2:**
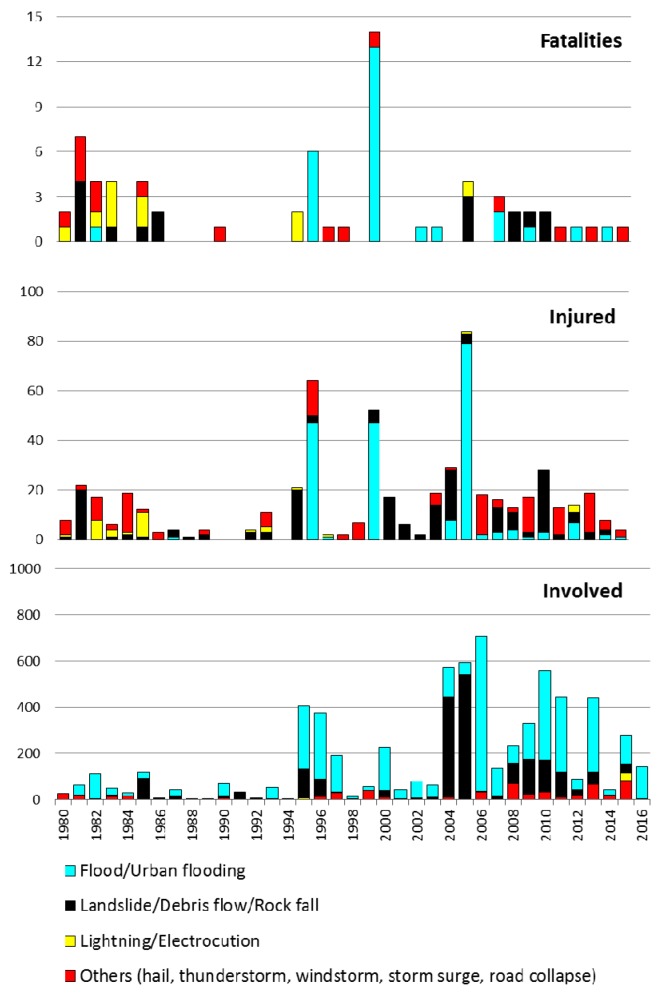
Yearly trend of fatalities, injured and involved people during the period 1980–2016.

Figure 2 presents the yearly values of fatalities, injured and involved people per year and sorted according to the damaging phenomenon. The yearly average is 1.84 for fatalities and increases to 15.30 for injured and to 179.84 for involved people (Table 4). To confirm the proportions between fatalities, injured and involved people, it can be noted that the three diagrams use different scales; tens of people for fatalities, hundreds for injured and thousands for people involved. To make the graphical representation suitable, the damaging phenomena were clustered into four groups as follows: (a) types directly related to water (floods and urban flooding); (b) types of mass movement (landslides, debris flows and rock falls); (c) lightning; and (d) hail, windstorm, storm surge and road collapse, which were merged into a single group, due to the low number of cases per year.

During 24 of the 37 analyzed years, at least one fatality occurred. According to their spatially diffuse effects, floods/urban flooding caused the greatest number of fatalities, followed by landslides and lightning. The highest value occurred in 2000 (13 fatalities), due to the so-called Soverato event, after the name of the municipality on the eastern coast where 13 people died because of a flood that affected a campsite. The second highest value (seven fatalities) pertains to 1981 and was caused by landslides/debris flows/rock falls and other phenomena. The third highest value was recorded in 1996, when river floods killed six people in the town of Crotone, on the east coast. The “low” number of fatalities is in accordance with the relative “not significant” [48] values characterizing developed countries, and does not present specific trends, except for fatalities caused by lightning, which decreased during the second half of the study period. This trend can be partially related to the progressive shift of the population from rural to urban areas, as also detected in the U.S. during the twentieth century [39].

Injured occurred in all but two years of the study period. The peaks of injured occurred during the events that caused the greatest number of fatalities, confirming that these were the most severe cases of the study period. Nevertheless, the figures were not in the same order; the largest number of injured pertains to a DHE that affected the town of Vibo Valentia, on the west coast, causing four fatalities in 2006. In this case, a series of small torrents inundated some urbanized areas, mainly causing injury in tourist structures. A debris flow, along one of these torrents, swept away and killed two men and a child who were traveling by car. In addition, a farmer was killed by lightning. The second largest number of injured pertains to the aforementioned Crotone event, and the third concerns the Soverato event.

Involved people were recorded during each year of the study period, and the figures increased during the most recent years. The increased data during the second half of the study period can be partially related to increasing attention of media on the effects of DHEs and to a better diffusion of news and information characterizing the most recent decades with respect to the 1980s.

### 5.2. Age and Gender

**Figure 3:**
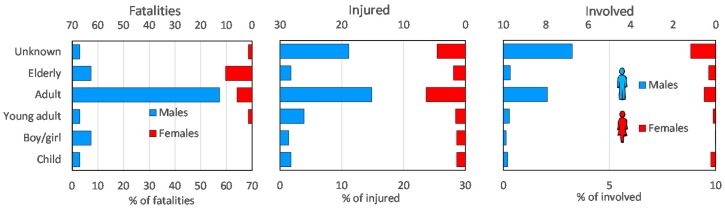
Age and gender of fatalities, injured and involved people (for the three diagrams, *Y* axis has the same legend reported on the first diagram).

The age was known for 95.59% of fatalities, 42.4% of injured and 15.04% of involved people (Table 5). The average age assessed based on the data available for each of the three groups and considering the gender (for the cases in which both gender and age were available) (Figure 3). Overall, the average age was highest for fatalities (46.90 years), medium for injured (37.87 years) and lowest for the involved people (34.80 years). This may be a result of greater mental alertness and the greater physical ability of young adults and adults, which may increase their chances to avoid (or survive) harmful events. This is confirmed by several cases of adults who could quickly swerve to avoid landslides or to rapidly abandon their flooded vehicle/house. Conversely, middle-aged and senior people injured themselves falling or suffered panic attacks when water, debris or mud suddenly entered their home/vehicle. The reliability of the data on gender is very high for fatalities, low for the injured (47.88% of cases unknown) and very low for involved people (91.31% of cases unknown). Taking into account that males currently represent 49% of the Calabrian population, it has been found that they were more numerous than females among the three groups of people. Based on cases in which the gender is known, the ratio of male/female is 4.4 for fatalities, 2 for injured and 2.6 for involved people. Considering classes of age and gender, male adults were more numerous among the three groups of people. Adult females were numerous in all the three groups, but, among fatalities, the elderly female class shows a slightly higher value.

Given the nature of the data sources used (newspapers), it is intrinsic to describe at least what occurred. For this reason, the type of phenomenon causing damage is known in all the cases, thus the reliability of this variable is very high for the three groups of affected people. The number and percentage of fatalities, injured and involved people sorted by type of phenomenon and gender are reported in Table 6. The type of phenomenon that physically affected the highest percentage of people during the study period was flood (31.61%), followed by landslide (29.39%) and urban flooding (27.09%).

Floods caused the highest values of fatalities (26; 38.24%), injured (194; 34.28%) and involved people (2084; 31.32%). The second most frequent phenomenon was landslides, causing 22.06% of the fatalities and 31.45% of the injured. For involved people, the second most represented phenomenon is urban flooding, causing 29.47% of cases. Males are more vulnerable than females in the case of landslides, lightning and road collapse. In fact, landslides and road collapse during the study period only killed males. Similarly, lightning killed mostly males (9 out of 10). Particularly, road collapse only affected males in all three groups. This occurred because, in the majority of cases, people that fell into a chasm opened on the road were workers driving heavy trucks, and, in Calabria, this activity is almost exclusively performed by males.

**Figure 4:**
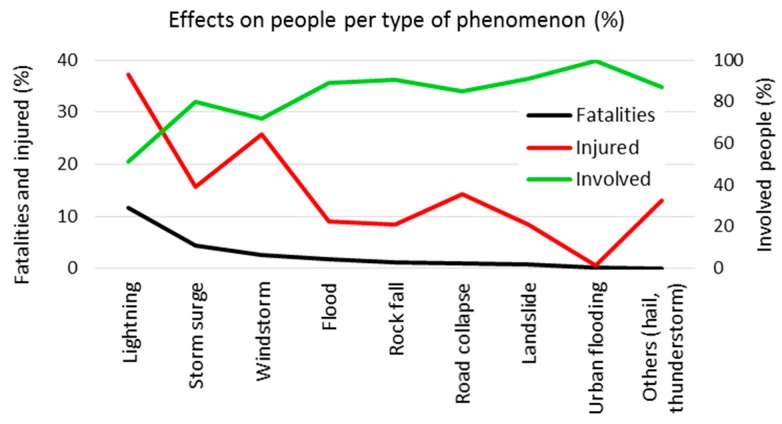
Percentage of fatalities, injured and involved people per type of phenomenon.

By comparing the number of fatalities, injured and involved people, a measure of the dangerousness of the different phenomena can be inferred (Figure 4). Actually, the most dangerous phenomena can be considered those that caused the highest percentage of fatalities among the total of people affected.

Accordingly, the most dangerous phenomenon is lightning; it affected 86 persons, representing a relatively low percentage of the total (1.18%), but nevertheless, a large percentage of them were killed (10, 11.63% of people affected by lightning) and injured (37.21%). Similarly, storm surges and windstorms affected a low number of people, causing high percentages of fatalities and injured. In contrast, urban flooding affected many people (27.09% of the total) but caused only one fatality.

**Figure 5:**
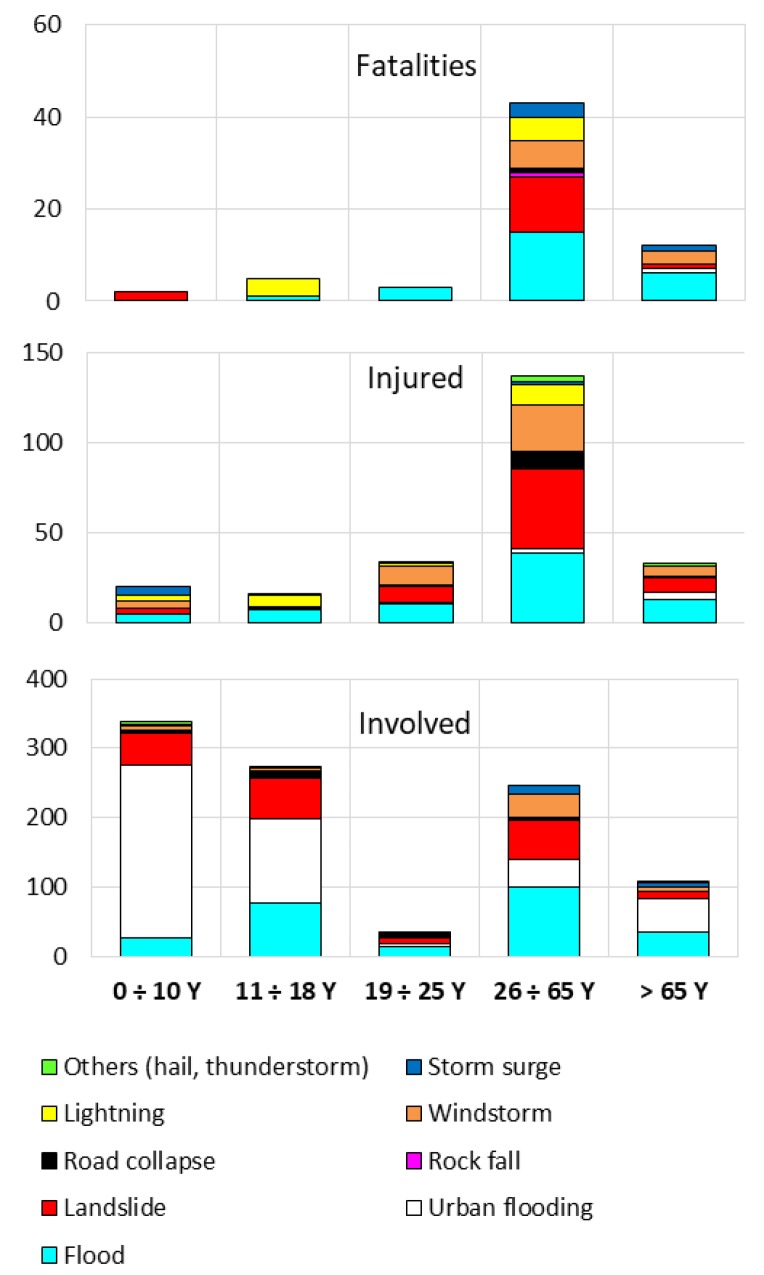
Relationships between age and type of phenomena (See also Appendix A: Table A1).

Figure 5 presents the ages of people affected by the different phenomena. A small percentage of fatalities is included in the first three classes, while the others are in the classes of 26 to 65 years and over 65 years. Urban flooding killed only a female in the elderly class. The injured are more uniformly distributed among the classes of age. The absence of injured by rock fall or road collapse under 18 years can be explained considering that this type of phenomenon occurs more frequently with drivers of vehicles, thus it is less probable for people under 18 years, who cannot drive in Italy. For involved people, the reliability of age is very low, but, for the cases in which this variable is known, people generally belong to all the classes of age.

### 5.3. Place, Condition, Activity and Dynamic

**Figure 6:**
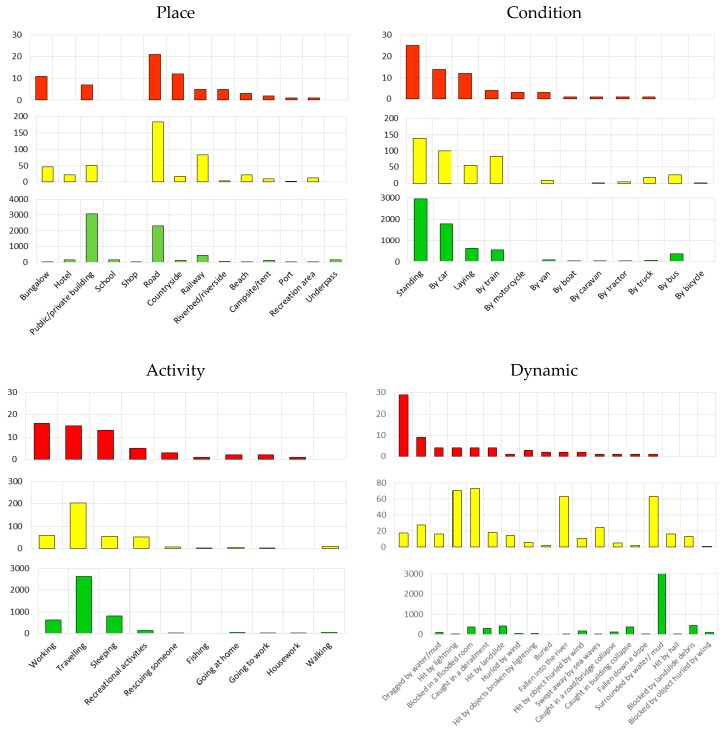
Place, condition, activity and dynamic for fatalities (**red**), injured (**yellow**) and involved people (**green**) (See also Appendix A: Table A2).

Data on place, condition, dynamic and activity are presented in Figure 6. The most frequent conditions of people affected were standing (42.8%), by car (25.96%) and lying (9.4%). Concerning the activity, traveling was the most common for the whole of the affected people (39.08%), followed by sleeping (12.1%) and working (9.7%), but there are some differences among the three groups. While traveling was the most common activity for both injured (35.69%) and involved people (39.54%), fatalities mainly occurred while working (23.53%). The most common dynamic for the whole of the affected people was to be surrounded by water/mud (57.96%). The greatest contribution depends on the 62.52% of people involved, followed by 11.13% of injured and only 1.47% of fatalities. In contrast, the dynamic of being dragged by water/mud caused the highest percentage of fatalities (42.65%) and low percentages of both injured (3.0%) and involved people (1.46%). From this comparison between fatalities, injured and involved people, one can conclude that being dragged by water/mud is the most dangerous dynamic; only a few people were involved without suffering damage or being injured, while the other people affected were killed. A similar path characterized the dynamic of being hit by lightning; relatively few people found themselves in this dynamic but a large percentage died. In contrast, being hit by hail, blocked by landslide debris or blocked by an object hurled by the wind did not cause fatalities, even if some were injured and involved. Thus, being dragged by water/mud and surrounded by water/mud represented the two extremes of dangerousness, namely, the dragging effect of rapid-flowing water totally/partially obstructed the attempts of people to save their lives, respectively. In contrast, the dynamic of being surrounded by water/mud was described as steady water in which people, on foot or by vehicle, were in difficulties but ultimately survived. When looking at the place-phenomena relationships, it can be seen that fatalities and injured occurred more frequently outdoors (73.53% and 58.48%, respectively) than indoors, while the majority of involved people were indoors (51.16%), in public and private buildings (23.56%) (Table 7). The combination of bungalow/floods caused 16.18% of fatalities but caused a low percentage of injured (8.13%) and involved people (0.2%). This is because all of the reported fatalities died during a single event (the Soverato flood). As a more general result, roads are the places where people are most frequently killed, in a combination of road/floods (11.76% of fatalities), road/landslides (10.29%) and road/windstorms (5.88%). In particular, landslides during the study period frequently injured people traveling on the regional roads (13.96%) and railways (14.31%).

### 5.4. Causes of Death and Types of Injures

**Figure 7:**
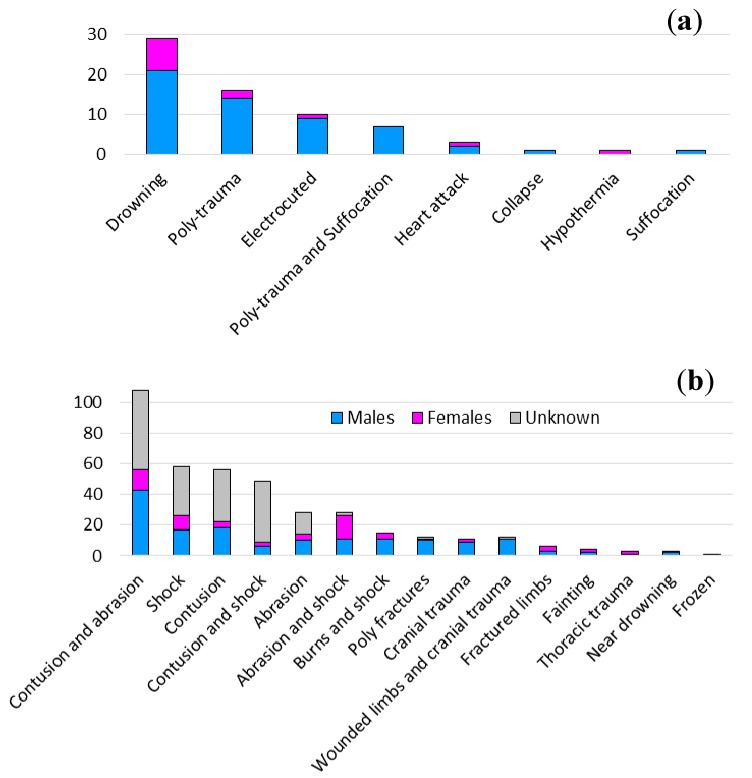
Gender, causes of death (**a**) and type of injured (**b**) (See also Appendix A: Table A3). The colours of the legend are the same for both diagrams.

Data on causes of death/types of injures and gender are reported in Figure 7. The medical cause of death is known for all the fatalities; drowning was the most frequent (42.65%) followed by poly-trauma (23.53%) and electrocution (14.71%). Among the injured, the type of injury is unknown for 30.39%; in the known cases, the majority of people suffered contusions and abrasions (19.08%) and shock (10.25%). Females were less numerous than males in each of the three groups. The only exceptions were for hypothermia, which killed only one woman, and abrasion and shock, affecting more females (2.65%) than males (1.94%).

Looking at the type of phenomenon, drowning/floods was the combination causing the majority of fatalities (35.29%), even if during floods, two people died due to collapse and hypothermia (Table 8). Windstorms caused fatalities both by poly-trauma (11.76%) and heart attack (1.47%). Landslides killed people by both poly-trauma (8.82%) and poly-trauma and suffocation (10.29%). Heart attacks caused two fatalities, in combination with urban flooding and storm surge. Overall, floods and landslides caused a similar percentage of injured people (34.28% and 31.45%, respectively), followed by windstorms (16.43%).

### 5.5. Hazardous and Protective Behaviors of People

A relatively small number of harmful (careless, negligent) or self-protective (defensive, safeguarding) behaviors adopted by individuals has been found and classified. We recognize that the amount of data collected is quite exiguous, but it was sufficient to classify the main types of behaviors.

Analyzing hazardous behavior and gender (Table 9), we found a prevalence of males among the three groups of affected people as follows:

(1) *Fatalities*: We found hazardous behavior for 13.24% of cases, concerning only males; the most frequent cases were being under a tree during lightning and fording or staying along rivers.
(2) *Injured:* Only three cases were recorded, two males and a female. Nevertheless, this does not exclude hazardous behaviors not reported by our data sources.
(3) *Involved people:* Hazardous behaviors were largely documented and performed more often by males than by females, even if, in this group of people, the gender is unknown in several cases. The most frequent circumstance was entering into a flooded underpass, which was detected in 121 cases, 19 involving males, three involving females, and the remaining 99 of unknown gender. Another frequent hazardous behavior was fording rivers, which was recorded in 23 cases, nine for males and six for females.

For *protective behaviors*, the ratio between males and females is still high in the three groups as follows:

(4) *Fatalities*: Protective behaviors were adopted by a relatively low number of persons; four males and one female died trying to save someone else, while another two males tried to save their lives climbing trees, and a female died trying to grab to her son.
(5) *Injured*: Trying to save someone was detected for four males and one female, respectively. To get out of from cars/trains is quite frequent, carried out in 33 cases by males.
(6) *Involved*: Even in this group, the group of people that tried to save someone else included 35 males and one female. The most diffuse behavior in this group was climbing on the roof/upper floor, which was recorded in more than one thousand cases at a similar percentage by males and females.

**Figure 8:**
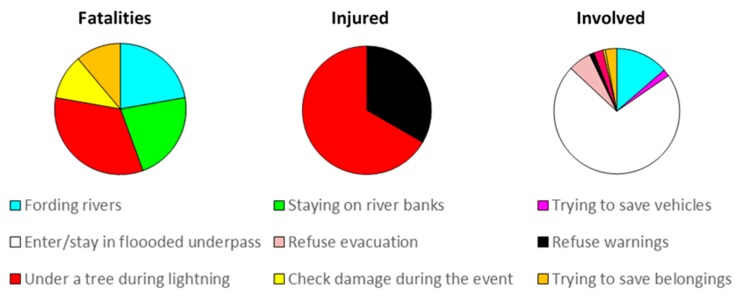
Damage severity and hazardous behaviors.

Figure 8 represents the relationships between *hazardous behavior* and damage severity, and Appendix A Table A4 reports the relationships between *hazardous behaviors* versus type of phenomenon.

(7) *Fatalities*: Lightning/staying under a tree during lightning was the reported behavior of three of the 10 lightning fatalities during the study period. Harmful behaviors were evident for floods and resulted in four fatalities. Two persons died fording a river, and two died staying on the riverbed. Behaviors regarding the concern of people for their properties caused two fatalities during storm surge.
(8) *Injured:* A few people were injured showed hazardous behaviors, one in the case of a windstorm and two in the case of lightning.
(9) *Involved*: In this group, the behaviors were mainly related to floods; in 23 cases fording rivers and in 121 entering flooded underpasses. Two of these persons were homeless sleeping in the underpass. It must be noted that, for landslides, the only hazardous behavior was to refuse evacuation, which was recorded for 10 involved persons.
(10) *Fatalities:* An attempt to rescue someone was recorded during flood, urban flooding, landslide, windstorm and storm surge. Flood caused four fatalities. Further protective behaviors were detected in flood fatalities, such as climbing on the car roof, grabbing on to someone, and climbing trees, even if these behaviors did not save their lives.
(11) *Injured*: The majority of injured people that exhibited a protective behavior get out from car/train hit by a landslide (33 cases).
(12) *Involved people*: The largest number involved people climbing on a roof/upper floor in the case of floods. During landslides, cases of protective behaviors were more numerous and differentiated than for every other phenomena, including moving to a safer place (514 cases), escaping from cars/train (65), driving to avoid danger (29) and escaping from buildings (24).

Table 10 presents the relationships between protective behaviors and type of phenomenon.

## 6. Discussion

During the 37-year period, in Calabria, approximately 200 people per year were in some way affected by DHEs; for every 100 people involved, approximately 10 were injured and one died. The circumstances in which the events developed are discussed as follows.

(1) *Phenomena*: The majority of people were affected by floods and landslides, and secondarily by other damaging phenomena. Floods were more dangerous than landslides, causing the largest percentage of fatalities and injured, while urban flooding affected several people but killed only one person. This finding cannot be compared to international inventories, because they generally focus on a single type of phenomenon (floods or landslides). Nevertheless, a comparison to the national catalogue of flood and landslide fatalities in Italy from 1960 to 2014 [25] found that, at the national scale, landslides killed more people than did floods, while, in Calabria, the opposite was true. Nevertheless, the most dangerous phenomenon, even if less common, was lightning, as it affected a small number of people but caused a relatively high number of fatalities and injured.
(2) *Age*. The average age decreased from fatalities to injured to involved people, suggesting that the greater mental readiness and higher physical ability of young adults and adults may increase their chances of avoiding harmful events, while middle-aged and senior people injured themselves falling due to water and mud, or suffered panic attacks when water or mud rapidly moved into their home or car.
(3) *Gender*. Our results are in accordance with those of other authors (Table 1), who detected a greater exposition/vulnerability of males who were more numerous in all three severity levels. The ratios of male/female are 4.4 for fatalities, 2.0 for injured, and 2.6 for involved people. Adult females are numerous in all three groups, but, among fatalities, the elderly female class shows a slight vulnerability. Males are more vulnerable than females in the case of landslides, lightning and road collapse.
(4) *Hazardous/protective behaviors*. Individuals who faced an unexpected landslide did not show any clear hazardous behaviors and instead adopted self-protective actions (mainly escaping very quickly), thus reducing the potential landslide impact. For floods, this result was the opposite; several clear and identified careless and negligent actions were detected.

## 7. Conclusions

The impact of damaging hydrogeological events on people in Calabria was classified into three severity levels as follows: fatalities, injured and people involved. The comparative analysis of people affected according to these levels highlighted the phenomena and circumstances causing either the maximum damage (fatalities) or no damage at all (involved people).

During the 37-year study period, approximately 200 people per year were in some way affected. Of every 100 people involved, approximately 10 were injured and one was killed. Floods physically affected the highest percentage of people, followed by landslides and urban flooding. In contrast to the national level [4], floods killed more people than did landslides, and landslides mainly affected people outdoors, along roads, while at the national scale, they mainly occurred in peoples’ houses. Lightning, though uncommon, is the most dangerous phenomenon, causing a relatively high number of fatalities and injured.

In accordance with the literature, males show a greater exposure/vulnerability in the three severity levels, and sometimes hazardous behaviors, such as fording rivers and driving in flooded underpasses. Adult females are numerous in all the three groups, but, among fatalities, the elderly female class shows a slightly higher value. Fatalities were older than both injured and involved people, and we attributed the difference to the greater mental alertness and higher physical ability of young adults/adults, while middle-aged/senior people injured themselves falling due to water and mud, or suffered panic attacks when water/mud moved into their home/car.

Being dragged by water/mud and surrounded by water/mud represented the two extremes of dynamic dangerousness, namely, the dragging effect of rapidly flowing water totally or the partial obstruction of the attempts of people to save their lives, respectively. In contrast, people on foot or in a motor vehicle surrounded by steady water/mud encountered difficulties but ultimately survived. Drowning/floods was the combination causing the majority of fatalities. Windstorms caused fatalities both by poly-trauma and heart attack, while landslides killed people by both poly-trauma and poly-trauma and suffocation.

The results of this analysis could be used to promote communication strategies and informational campaigns aiming to bridge the gap between perception and actual risk conditions, and for designing recommendations for self-protecting actions and proactive policies that can contribute to a decrease in harm to the people of Calabria.

## Figures and Tables

**Table 1:** Review of papers containing data on flood fatalities.

Reference	Period	Country	Fatalities #	Male %	Female % ^1^	Affected Age (Years)
[18]	1788–1996	Australia	2213	80	20	<21; >70
[21]	1959–2005	USA	4586	Majority	Minority	10–19; >60
[22]	1997–2008	Australia	73	71.2	28.8	10–29; >70
[23]	1990–2008	South Korea	966	Majority	Minority	-
[16]	1980–2009	World	539,811	-	-	-
[24]	1959–2008	Texas	286	62.9	37.1	<30
[25]	1975–2002	World	175,864	-	-	-
[13]	1900–2015	Australia	1859	79	21	Children; <29
[19]	1865–2010	Portugal	1012	57.3	25.3	-
[14]	1970–2010	Greece	151	62.9	31.1	>35
[12]	1946–2015	Switzerland	124	75	25	0–9
[26]	2002–2012	Australia	129	64	36	55–64
[20]	1965–2014	Italy	771	57.6	42.4	20–89

^1^ If the sum of males and females is not 100, it means that some of the victims were of unknown gender. # Number.

**Table 2:** Review of papers containing data on landslide fatalities.

Reference	Period	Region/Country	Landslides #	Fatalities #
[28]	1840–1996	Canada	84	545
[29]	2001–2004	China	400	3000
[30]	1978–2005	Nepal	397	2179
[28]	2004–2010	World	2620	32,322
[31]	1950–2011	World	213	77,779
[32]	2004–2013	Latin America, Caribbean	611	11,631
[19]	1865–2010	Portugal	281	236
[33]	1995–2014	Europe	476	1370
[12]	1946–2015	Switzerland	-	-
[20]	1965–2014	Italy	405	1292

# Number.

**Table 3:** Structure of PEOPLE database.

**Event Identification**			
**Time of the Event**	**Type of Phenomenon**	**Victim Identification**	**Damage Severity**
Year (YYYY) Month (MM) Day (DD) ID (YYYY, MM, DD, #)	Flood Urban flooding Landslide Rock fall Road collapse Windstorm Lightning Storm surge Others (hail, thunderstorm)	Name Surname Gender Age	Fatalities Injured Involved people
**Victim-Event Interaction**			
**Place**	**Condition**	**Activity**	**Dynamic**
Indoor Public/private building Bungalow School Hotel Shop Outdoor Road Railway Riverbed/riverside Campsite/tent Underpass Beach Countryside Port Recreation area	Standing Laying By bicycle By motorcycle By car By bus By van By boat By caravan By tractor By truck By train	Working Traveling Sleeping Recreational activities Rescuing someone Fishing Going at home Going to work Housework Walking	Dragged by water/mud Hit by lightning Blocked in a flooded room Caught in a derailment Hit by landslide Hurled by wind Hit by objects broken by lightning Buried Fallen into the river Hit by object hurled by wind Swept away by sea waves Caught in a road/bridge collapse Caught in building collapse Fallen down a slope Surrounded by water/mud Hit by hail Blocked by landslide debris Blocked by object hurled by wind
**Effects on People**			
**Causes of Death**		**Types of Injuries**	
Drowning Poly-trauma Electrocuted Poly-trauma and suffocation Heart attack Collapse Hypothermia Suffocation		Contusion and abrasion Shock Contusion Contusion and shock Abrasion Abrasion and shock Burns and shock Poly fractures Cranial trauma Wounded limbs and cranial trauma Fractured limbs Fainting Thoracic trauma Near drowning Frozen	

# Number.

**Table 4:** Data reliability.

**Variable**	**Value**					
#PEO	7288					
#EV	740					
Average #PEO per event	9.85					
Average #PEO per year	196.97					
**Variable**	**Fatalities**		**Injured**		**Involved**	
	**#**	**%**	**#**	**%**	**#**	**%**
#PEO	68	*0.93*	566	*7.77*	6654	*91.30*
#EV	44	*5.95*	153	*20.68*	631	*85.27*
Average #PEO per event	1.55		3.70		10.55	
Average #PEO per year	1.84		15.30		179.84	
**Gender**						
Known cases	68	*100*	295	*52.12*	578	*8.69*
Unknown cases	0	*-*	271	*47.88*	6076	*91.31*
*Reliability*	*Very high*		*Low*		*Very low*	
**Age**						
Known cases	65	*95.59*	240	*42.40*	1001	*15.04*
Unknown cases	3	*4.41*	326	*57.60*	5653	*84.96*
*Reliability*	*Very high*		*Low*		*Very low*	
**Place**						
Known cases	68	*100*	449	*79.33*	6615	*99.41*
Unknown cases	0	*-*	117	*20.67*	39	*0.59*
*Reliability*	*Very high*		*High*		*Very high*	
**Condition**						
Known cases	65	*95.59*	434	*76.68*	6430	*96.63*
Unknown cases	3	*4.41*	132	*23.32*	224	*3.37*
*Reliability*	*Very high*		*Medium*		*Very high*	
**Activity**						
Known cases	58	*85.29*	394	*69.61*	4342	*65.25*
Unknown cases	10	*14.71*	172	*30.39*	2312	*34.75*
*Reliability*	*High*		*Medium*		*Medium*	
**Dynamic**						
Known cases	68	*100*	441	*77.92*	6615	*99.41*
Unknown cases	0	*-*	125	*22.08*	39	*0.59*
*Reliability*	*Very high*		*Medium*		*Very high*	
**Variable Reliability**	(% unknown cases)					
*Very high*	*0*–*5%*					
*High*	*5.1*–*20%*					
*Medium*	*20.1*–*40%*					
*Low*	*40.1*–*60%*					
*Very low*	*>60%*					

#: number; PEO: people; EV: events; percentages in italics.

**Table 5:** Age and gender of fatalities, injured and involved people.

Variable		Fatalities						Injured								Involved							
		68						566								6654							
		Total Known		Males		Females		Total Known		Males		Females		Unknown		Total Known		Males		Females		Unknown	
		#	*%*	#	*%*	#	%	#	%	#	%	#	%	#	%	#	%	#	%	#	%	#	%
Known age		65	*95.59*	53	*77.94*	12	*17.65*	240	*42.40*	134	*23.67*	72	*12.72*	34	*6.01*	1001	*15.04*	202	*3.04*	82	*1.23*	717	*10.78*
Known gender		68	*100.00*	55	*80.88*	13	*19.12*	295	*52.12*	197	*34.81*	98	*17.31*	271	*47.88*	578	*8.69*	418	*6.28*	160	*2.40*	6076	*91.31*
Average age		46.90		42.86		64.08		37.87		38.18		37.31		-	-			34.80	34.73	37.55		17.67	
	Years																						
Child	0–10	2	*2.94*	2	*2.94*	-	*-*	20	*3.53*	10	*1.77*	8	*1.41*	2	*0.35*	338	*5.08*	14	*0.21*	15	*0.23*	309	*4.64*
Boy/girl	11–18	5	*7.35*	5	*7.35*	-	*-*	16	*2.83*	8	*1.41*	8	*1.41*	-	*-*	274	*4.12*	9	*0.14*	1	*0.02*	264	*3.97*
Young adult	19–25	3	*4.41*	2	*2.94*	1	*1.47*	34	*6.01*	22	*3.89*	9	*1.59*	3	*0.53*	34	*0.51*	19	*0.29*	8	*0.12*	7	*0.11*
Adult	26–65	43	*63.24*	39	*57.35*	4	*5.88*	137	*24.20*	84	*14.84*	36	*6.36*	17	*3.00*	246	*3.70*	138	*2.07*	36	*0.54*	72	*1.08*
Elderly	>65	12	*17.65*	5	*7.35*	7	*10.29*	33	*5.83*	10	*1.77*	11	*1.94*	12	*2.12*	109	*1.64*	22	*0.33*	22	*0.33*	65	*0.98*
Unknown	Unknown	3	*4.41*	2	*2.94*	1	*1.47*	326	*57.60*	63	*11.13*	26	*4.59*	237	*41.87*	5653	*84.96*	216	*3.25*	78	*1.17*	5359	*80.54*

#: Nuzmber; percentages in italics.

**Table 6:** Relationship between type of phenomenon and gender.

Phenomenon	Total People		Fatalities						Injured								Involved							
			# Fatalities		Males		Females		# Injured		Males		Females		Unknown		# Involved		Males		Females		Unknown	
	#	%	#	*%*	#	*%*	#	*%*	#	*%*	#	*%*	#	*%*	#	*%*	#	*%*	#	*%*	#	*%*	#	*%*
Flood	2304	*31.61*	26	*38.24*	17	*25.00*	9	*13.24*	194	*34.28*	48	*8.48*	42	*7.42*	104	*18.37*	2084	*31.32*	126	*1.89*	60	*0.90*	1898	*28.52*
Urban flooding	1974	*27.09*	1	*1.47*	-	-	1	*1.47*	12	*2.12*	3	*0.53*	3	*0.53*	6	*1.06*	1961	*29.47*	63	*0.95*	38	*0.57*	1860	*27.95*
Landslide	2142	*29.39*	15	*22.06*	15	*22.06*	-	-	178	*31.45*	64	*11.31*	26	*4.59*	88	*15.55*	1949	*29.29*	127	*1.91*	41	*0.62*	1781	*26.77*
Rock fall	55	*0.75*	1	*1.47*	1	*1.47*	-	-	5	*0.88*	2	*0.35*	1	*0.18*	2	*0.35*	49	*0.74*	11	*0.17*	4	*0.06*	34	*0.51*
Road collapse	112	*1.54*	1	*1.47*	1	*1.47*	-	-	16	*2.83*	16	*2.83*	-	-	-	-	95	*1.43*	19	*0.29*	-	-	76	*1.14*
Windstorm	361	*4.95*	9	*13.24*	7	*10.29*	2	*2.94*	93	*16.43*	31	*5.48*	11	*1.94*	51	*9.01*	259	*3.89*	40	*0.60*	6	*0.09*	213	*3.20*
Lightning	86	*1.18*	10	*14.71*	9	*13.24*	1	*1.47*	32	*5.65*	24	*4.24*	8	*1.41*	-	-	44	*0.66*	4	*0.06*	2	*0.03*	38	*0.57*
Storm surge	115	*1.58*	5	*7.35*	5	*7.35*	-	-	18	*3.18*	6	*1.06*	6	*1.06*	6	*1.06*	92	*1.38*	27	*0.41*	9	*0.14*	56	*0.84*
Others (hail, thunderstorm)	139	*1.91*	-	-	-	-	-	-	18	*3.18*	3	*0.53*	1	*0.18*	14	*2.47*	121	*1.82*	1	*0.02*	-	-	120	*1.80*

#: Nuzmber; percentages in italics.

**Table 7:** Fatalities, injured and involved according to type of phenomenon and place.

**Fatalities**																					
**Total Fatalities**				**Flood**		**Urban Flooding**		**Landslide**		**Rock Fall**		**Road Collapse**		**Windstorm**		**Lightning**		**Storm Surge**		**Others (Hail, Thunderstorm)**	
**#**	***%***			**#**	***%***	**#**	***%***	**#**	***%***	**#**	***%***	**#**	***%***	**#**	***%***	**#**	***%***	**#**	***%***	**#**	***%***
18	*26.47*	Indoor	Bungalow	11	*16.18*	0	*-*	0	*-*	0	*-*	0	*-*	0	*-*	0	*-*	0	*-*	0	*-*
			Hotel	0	*-*	0	*-*	0	*-*	0	*-*	0	*-*	0	*-*	0	*-*	0	*-*	0	*-*
			Public/private building	0	*-*	1	*1.47*	1	*1.47*	0	*-*	0	*-*	2	*2.94*	2	*2.94*	1	*1.47*	0	*-*
50	*73.53*	Outdoor	Road/Bridge/Street	8	*11.76*	0	*-*	7	*10.29*	1	*1.47*	1	*1.47*	4	*5.88*	0	*-*	0	*-*	0	*-*
			Countryside	2	*2.94*	0	*-*	2	*2.94*	0	*-*	0	*-*	1	*1.47*	7	*10.29*	0	*-*	0	*-*
			Railway	0	*-*	0	*-*	4	*5.88*	0	*-*	0	*-*	1	*1.47*	0	*-*	0	*-*	0	*-*
			Riverbed/riverside	4	*5.88*	0	*-*	1	*1.47*	0	*-*	0	*-*	0	*-*	0	*-*	0	*-*	0	*-*
			Beach	0	*-*	0	*-*	0	*-*	0	*-*	0	-	0	*-*	0	-	3	*4.41*	0	-
			Campsite/tent	1	*1.47*	0	*-*	0	*-*	0	*-*	0	-	1	*1.47*	0	-	0	*-*	0	-
			Port	0	*-*	0	*-*	0	*-*	0	*-*	0	-	0	*-*	0	-	1	*1.47*	0	-
			Recreation area	0	*-*	0	*-*	0	*-*	0	-	0	-	0	*-*	1	1.47	0	*-*	0	-
			Not reported	0	*-*	0	*-*	0	*-*	0	*-*	0	-	0	*-*	0	-	0	*-*	0	-
**Injured**																					
**Total Injured**				**Flood**		**Urban Flooding**		**Landslide**		**Rock Fall**		**Road Collapse**		**Windstorm**		**Lightning**		**Storm Surge**		**Others (Hail, Thunderstorm)**	
**#**	***%***			**#**	***%***	**#**	***%***	**#**	***%***	**#**	***%***	**#**	***%***	**#**	***%***	**#**	***%***	**#**	***%***	**#**	***%***
118	*20.85*	Indoor	Bungalow	46	*8.13*	0	*-*	0	*-*	0	*-*	0	*-*	0	*-*	0	*-*	0	*-*	0	*-*
			Hotel	20	*3.53*	1	*0.18*	0	*-*	0	*-*	0	*-*	0	*-*	0	*-*	0	*-*	0	*-*
			Public/private building	14	*2.47*	4	*0.71*	13	*2.30*	2	*0.35*	0	*-*	8	*1.41*	8	*1.41*	2	*0.35*	0	*-*
331	*58.48*	Outdoor	Road/Bridge/Street	15	*2.65*	6	*1.06*	79	*13.96*	2	*0.35*	15	*2.65*	53	*9.36*	4	*0.71*	4	*0.71*	5	*0.88*
			Countryside	1	*0.18*	0	*-*	3	*0.53*	0	*-*	0	*-*	5	*0.88*	6	*1.06*	0	*-*	2	*0.35*
			Railway	0	*-*	0	*-*	81	*14.31*	0	*-*	0	*-*	2	*0.35*	0	*-*	0	*-*	0	*-*
			Riverbed/riverside	2	*0.35*	0	*-*	1	*0.18*	0	*-*	1	*0.18*	0	*-*	0	*-*	0	*-*	0	*-*
			Beach	0	*-*	0	-	1	0.18	1	0.18	0	-	8	*1.41*	1	0.18	10	*1.77*	0	-
			Campsite/tent	1	*0.18*	0	-	0	-	0	-	0	-	8	*1.41*	0	-	0	*-*	0	-
			Port	0	*-*	0	-	0	-	0	-	0	-	0	*-*	0	-	2	*0.35*	0	-
			Recreation area	0	*-*	0	-	0	-	0	-	0	-	0	*-*	12	2.12	0	*-*	0	-
117	*20.67*		Not reported	95	*16.78*	1	0.18	0	-	0	-	0	-	9	*1.59*	1	0.18	0	*-*	11	1.94
**Involved**																					
**Total Involved**				**Flood**		**Urban Flooding**		**Landslide**		**Rock Fall**		**Road Collapse**		**Windstorm**		**Lightning**		**Storm Surge**		**Others (Hail, Thunderstorm)**	
**#**	***%***			**#**	***%***	**#**	***%***	**#**	***%***	**#**	***%***	**#**	***%***	**#**	***%***	**#**	***%***	**#**	***%***	**#**	***%***
3404	*51.16*	Indoor	Bungalow	13	*0.20*	0	*-*	0	*-*	0	*-*	0	*-*	0	*-*	0	*-*	0	*-*	0	*-*
			Hotel	140	*2.10*	0	*-*	1	*0.02*	0	*-*	0	*-*	0	*-*	0	*-*	0	*-*	0	*-*
			Public/private building	1568	*23.56*	692	*10.40*	677	*10.17*	4	*0.06*	0	*-*	49	*0.74*	32	*0.48*	40	*0.60*	8	*0.12*
			School	0	*-*	160	*2.40*	0	*-*	0	*-*	0	*-*	0	*-*	0	*-*	0	*-*	0	*-*
			Shop	0	*-*	0	*-*	0	*-*	0	*-*	0	*-*	0	*-*	0	*-*	20	*0.30*	0	*-*
3211	*48.26*	Outdoor	Road/Bridge/Street	249	*3.74*	884	*13.29*	719	*10.81*	39	*0.59*	95	*1.43*	207	*3.11*	8	*0.12*	6	*0.09*	109	*1.64*
			Countryside	20	*0.30*	0	*-*	93	*1.40*	0	*-*	0	*-*	2	*0.03*	2	*0.03*	0	*-*	4	*0.06*
			Railway	0	*-*	0	*-*	438	*6.58*	0	*-*	0	*-*	1	*0.02*	0	*-*	0	*-*	0	*-*
			Riverbed/riverside	37	*0.56*	0	*-*	3	*0.05*	0	*-*	0	*-*	0	*-*	0	*-*	0	*-*	0	*-*
			Beach	0	*-*	0	-	0	-	6	0.09	0	-	0	*-*	0	-	19	*0.29*	0	-
			Campsite/tent	25	*0.38*	100	1.50	0	-	0	-	0	-	0	*-*	0	-	0	*-*	0	-
			Port	0	*-*	0	-	0	-	0	-	0	-	0	*-*	0	-	4	*0.06*	0	-
			Recreation area	0	*-*	0	-	0	-	0	-	0	-	0	*-*	2	0.03	2	*0.03*	0	-
			Underpass/Tunnel	0	*-*	121	1.82	16	0.24	0	-	0	-	0	*-*	0	-	0	*-*	0	-
39	*0.59*		Not reported	32	*0.48*	4	0.06	2	0.03	0	-	0	-	0	*-*	0	-	1	*0.02*	0	-

#: Number; percentages in italics.

**Table 8:** Causes of death and types of injures according to type of phenomenon.

**Cause of Death**	**Flood**		**Urban Flooding**		**Landslide**		**Rock Fall**		**Road Collapse**		**Windstorm**		**Lightning**		**Storm Surge**		**Others (Hail, Thunderstorm)**	
	**#**	***%***	**#**	***%***	**#**	***%***	**#**	***%***	**#**	***%***	**#**	***%***	**#**	***%***	**#**	***%***	**#**	***%***
**Total**	**26**	***38.2***	**1**	***1.47***	**15**	***22.06***	**1**	***1.47***	**1**	***1.47***	**9**	***13.2***	**10**	***14.7***	**5**	***7.35***	**0**	***0***
Drowning	24	*35.29*	0	*-*	1	*1.47*	0	*-*	0	*-*	0	*-*	0	*-*	4	*5.88*	0	*-*
Poly-trauma	0	*-*	0	*-*	6	*8.82*	1	*1.47*	1	*1.47*	8	*11.76*	0	*-*	0	*-*	0	*-*
Electrocuted	0	*-*	0	*-*	0	*-*	0	*-*	0	*-*	0	*-*	10	*14.71*	0	*-*	0	*-*
Poly-trauma and Suffocation	0	*-*	0	*-*	7	*10.29*	0	*-*	0	*-*	0	*-*	0	*-*	0	*-*	0	*-*
Heart attack	0	*-*	1	*1.47*	0	*-*	0	*-*	0	*-*	1	*1.47*	0	*-*	1	*1.47*	0	*-*
Collapse	1	*1.47*	0	*-*	0	*-*	0		0	*-*	0	*-*	0	*-*	0	*-*	0	*-*
Hypothermia	1	*1.47*	0	*-*	0	*-*	0	*-*	0	*-*	0	*-*	0	*-*	0	*-*	0	*-*
Suffocation	0	*-*	0	*-*	1	*1.47*	0	*-*	0	*-*	0	*-*	0	*-*	0	*-*	0	*-*
Unknown	0	*-*	0	*-*	0	*-*	0	*-*	0	*-*	0	*-*	0	*-*	0	*-*	0	*-*
**Type of Injury**	**Flood**		**Urban Flooding**		**Landslide**		**Rock Fall**		**Road Collapse**		**Windstorm**		**Lightning**		**Storm Surge**		**Others (Hail, Thunderstorm)**	
	**#**	***%***	**#**	***%***	**#**	***%***	**#**	***%***	**#**	***%***	**#**	***%***	**#**	***%***	**#**	***%***	**#**	***%***
**Total**	**194**	***34.3***	**12**	***2.12***	**178**	***31.45***	**5**	***0.88***	**16**	***2.83***	**93**	***16.4***	**32**	***5.65***	**18**	***3.18***	**18**	***3.18***
Contusion and abrasion	5	*0.88*	0	*-*	45	*7.95*	0	*-*	1	*0.18*	49	*8.66*	0	*-*	7	*1.24*	1	*0.18*
Shock	14	*2.47*	9	*1.59*	22	*3.89*	0	*-*	0	*-*	0	*-*	13	*2.30*	0	*-*	0	*-*
Contusion	0	*-*	0	*-*	29	*5.12*	4	*0.71*	1	*0.18*	18	*3.18*	1	*0.18*	0	*-*	3	*0.53*
Contusion and shock	6	*1.06*	0	*-*	27	*4.77*	0	*-*	1	*0.18*	11	*1.94*	0	*-*	4	*0.71*	0	*-*
Abrasion	2	*0.35*	0	*-*	6	*1.06*	0	*-*	6	*1.06*	1	*0.18*	0	*-*	0	*-*	13	*2.30*
Abrasion and shock	1	*0.18*	1	*0.18*	22	*3.89*	0	*-*	3	*0.53*	0	*-*	0	*-*	1	*0.18*	0	*-*
Burns and shock	0	*-*	0	*-*	0	*-*	0	*-*	0	*-*	0	*-*	15	*2.65*	0	*-*	0	*-*
Poly fractures	2	*0.35*	1	*0.18*	7	*1.24*	0	*-*	0	*-*	2	*0.35*	0	*-*	0	*-*	0	*-*
Cranial trauma	0	*-*	1	*0.18*	2	*0.35*	0	*-*	1	*0.18*	7	*1.24*	0	*-*	0	*-*	0	*-*
Wounded limbs and cranial trauma	0	*-*	0	*-*	6	*1.06*	0	*-*	3	*0.53*	2	*0.35*	1	*0.18*	0	*-*	0	*-*
Fractured limbs	0	*-*	0	*-*	4	*0.71*	0	*-*	0	*-*	1	*0.18*	1	*0.18*	0	*-*	0	*-*
Fainting	1	*0.18*	0	*-*	0	*-*	0	*-*	0	*-*	1	*0.18*	0	*-*	2	*0.35*	0	*-*
Thoracic trauma	0	*-*	0	*-*	1	*0.18*	1	*0.18*	0	*-*	0	*-*	1	*0.18*	0	*-*	0	*-*
Near drowning	0	*-*	0	*-*	0	*-*	0	*-*	0	*-*	0	*-*	0	*-*	3	*0.53*	0	*-*
Frozen	0	*-*	0	*-*	0	*-*	0	*-*	0	*-*	0	*-*	0	*-*	0	*-*	1	*0.18*
Unknown	163	*28.80*	0	*-*	7	*1.24*	0	*-*	0	*-*	1	*0.18*	0	*-*	1	*0.18*	0	*-*

#: Number; percentages in italics.

**Table 9:** Gender and hazardous and protective behaviors.

	**Fatalities**						**Injured**								**Involved**							
**Hazardous Behavior**	**Total**		**Males**		**Females**		**Total**		**Males**		**Females**		**Unknown**		**Total**		**Males**		**Females**		**Unknown**	
	**#**	***%***	**#**	***%***	**#**	***%***	**#**	***%***	***#***	***%***	**#**	***%***	**#**	***%***	***#***	***%***	**#**	***%***	**#**	***%***	***#***	***%***
Under a tree during lightning	3	*4.41*	3	*4.41*	0	*-*	2	*0.35*	*1*	*0.18*	1	*0.18*	0	*-*	*4*	*0.06*	3	*0.05*	1	*0.02*	*0*	*-*
Fording rivers	2	*2.94*	2	*2.94*	0	*-*	0	*-*	*0*	*-*	0	*-*	0	*-*	*23*	*0.35*	9	*0.14*	6	*0.09*	*8*	*0.12*
Check damage during the event	1	*1.47*	1	*1.47*	0	*-*	0	*-*	*0*	*-*	0	*-*	0	*-*	*1*	*0.02*	0	*-*	1	*0.02*	*0*	*-*
Staying on river banks	2	*2.94*	2	*2.94*	0	*-*	0	*-*	*0*	*-*	0	*-*	0	*-*	*0*	*-*	0	*-*	0	*-*	*0*	*-*
Trying to save belongings	1	*1.47*	1	*1.47*	0	*-*	0	*-*	*0*	*-*	0	*-*	0	*-*	*5*	*0.08*	4	*0.06*	1	*0.02*	*0*	*-*
Refuse evacuation	0	*-*	0	*-*	0	*-*	0	*-*	*0*	*-*	0	*-*	0	*-*	*10*	*0.15*	1	*0.02*	1	*0.02*	*8*	*0.12*
Refuse warnings	0	*-*	0	*-*	0	*-*	1	*0.18*	*1*	*0.18*	0	*-*	0	*-*	*2*	*0.03*	2	*0.03*	0	*-*	*0*	*-*
Trying to save vehicles	0	*-*	0	*-*	0	*-*	0	*-*	*0*	*-*	0	*-*	0	*-*	*3*	*0.05*	3	*0.05*	0	*-*	*0*	*-*
Enter in a floooded underpass	0	*-*	0	*-*	0	*-*	0	*-*	*0*	*-*	0	*-*	0	*-*	*121*	*1.82*	19	*0.29*	3	*0.05*	*99*	*1.49*
Not reported	59	*86.76*	46	*67.65*	13	*19.12*	563	*99.47*	*195*	*34.45*	97	*17.14*	271	*47.88*	*6485*	*97.46*	377	*5.67*	147	*2.21*	*5961*	*89.59*
**Protective Behavior**	**Total**		**Males**		**Females**		**Total**		**Males**		**Females**		**Unknown**		**Total**		**Males**		**Females**		**Unknown**	
Rescuing someone	4	*5.88*	3	*4.41*	1	*1.47*	5	*0.88*	*4*	*0.71*	1	*0.18*	0	*-*	*41*	*0.62*	35	*0.53*	1	*0.02*	*5*	*0.08*
Climbing trees	2	*2.94*	2	*2.94*	0	*-*	3	*0.53*	*2*	*0.35*	1	*0.18*	0	*-*	*0*	*-*	0	*-*	0	*-*	*0*	*-*
Grabbing on to someone/something	2	*2.94*	1	*1.47*	1	*1.47*	5	*0.88*	*3*	*0.53*	2	*0.35*	0	*-*	*6*	*0.09*	5	*0.08*	1	*0.02*	*0*	*-*
Getting on the car roof	1	*1.47*	1	*1.47*	0	*-*	0	*-*	*0*	*-*	0	*-*	0	*-*	*21*	*0.32*	3	*0.05*	1	*0.02*	*17*	*0.26*
Break strustures to allow flood streaming	0	*-*	0	*-*	0	*-*	0	*-*	*0*	*-*	0	*-*	0	*-*	*1*	*0.02*	0	*-*	1	*0.02*	*0*	*-*
Building a temporary dam	0	*-*	0	*-*	0	*-*	0	*-*	*0*	*-*	0	*-*	0	*-*	*36*	*0.54*	4	*0.06*	2	*0.03*	*30*	*0.45*
Driving to avoid danger	0	*-*	0	*-*	0	*-*	5	*0.88*	*2*	*0.35*	1	*0.18*	2	*0.35*	*45*	*0.68*	23	*0.35*	0	*-*	*22*	*0.33*
Getting on roof/upper floor	0	*-*	0	*-*	0	*-*	10	*1.77*	*3*	*0.53*	3	*0.53*	4	*0.71*	*1114*	*16.74*	13	*0.20*	14	*0.21*	*1087*	*16.34*
Getting out of buildings	0	*-*	0	*-*	0	*-*	1	*0.18*	*0*	*-*	1	*0.18*	0	*-*	*134*	*2.01*	4	*0.06*	3	*0.05*	*127*	*1.91*
Getting out of cars/train	0	*-*	0	*-*	0	*-*	33	*5.83*	*2*	*0.35*	0	*-*	31	*5.48*	*138*	*2.07*	20	*0.30*	5	*0.08*	*113*	*1.70*
Moving to safer place	0	*-*	0	*-*	0	*-*	2	*0.35*	*0*	*-*	1	*0.18*	1	*0.18*	*584*	*8.78*	22	*0.33*	13	*0.20*	*549*	*8.25*
Swimming/Swimming in flooded rooms	0	*-*	0	*-*	0	*-*	6	*1.06*	*4*	*0.71*	2	*0.35*	0	*-*	*10*	*0.15*	6	*0.09*	2	*0.03*	*2*	*0.03*
To use a track to save people	0	*-*	0	*-*	0	*-*	0	*-*	*0*	*-*	0	*-*	0	*-*	*8*	*0.12*	4	*0.06*	0	*-*	*4*	*0.06*
Not reported	59	*86.76*	48	*70.59*	11	*16.18*	496	*87.63*	*177*	*31.27*	86	*15.19*	233	*41.17*	*4516*	*67.87*	279	*4.19*	117	*1.76*	*4120*	*61.92*

#: Number; percentages in italics.

**Table 10:** Types of phenomena and protective behaviors.

Phenomenon	Protective Behavior	Fatalities	Injured	Involved	Total
		#	#	#	#
Flood	Rescuing someone	4	4	17	25
Urban flooding				7	7
Landslide				10	10
Windstorm				3	3
Storm surge			1	4	5
Flood	Getting out of buildings		1	39	40
Urban flooding				49	49
Landslide				24	24
Storm surge				20	20
Others				2	2
Flood	Getting out of cars/train			22	22
Urban flooding				32	32
Landslide			33	65	98
Road collapse				17	17
Storm surge				2	2
Landslide	Driving to avoid danger		5	29	34
Rock fall				7	7
Road collapse				6	6
Windstorm				1	1
Lightning				1	1
Others				1	1
Flood	Moving to safer place		1	45	46
Urban flooding				2	2
Landslide			1	514	515
Rock fall				2	2
Windstorm				17	17
Others				4	4
Flood	Getting on the car roof	1		4	5
Urban flooding				17	17
Flood	Getting on roof/upper floor		10	1036	1046
Urban flooding				70	70
Landslide				8	8
Flood	Grabbing on to someone/something	2	2	4	8
Landslide			3	1	4
Storm surge				1	1
Flood	To use a track to save people			2	2
Urban flooding				5	5
Storm surge				1	1
Flood	Climbing trees	2	3		5
Flood	Break structures to allow flood streaming			1	1
Flood	Building a temporary dam			25	25
Urban flooding				7	7
Landslide				4	4
Flood	Swimming (in flooded rooms or grabbed to something)		5	4	9
Urban flooding				3	3
Landslide			1	3	4
Total		9	70	2138	2217

#: Number.

## Appendix A

**Table A1:** Relationships between classes of age and types of phenomenon.

**Fatalities**														
**Type of Phenomenon**	**Total Fatalities**		**0****–****10 Y**		**11****–****18 Y**		**19****–****25 Y**		**26****–****65 Y**		**>65 Y**		**Unknown**	
	**#**	***%***	**#**	***%***	**#**	***%***	**#**	***%***	**#**	***%***	**#**	***%***	**#**	***%***
Flood	26	*37.14*	-	*-*	1	*1.43*	3	*4.29*	15	*21.43*	6	*8.57*	1	*1.43*
Urban flooding	1	*1.43*	-	*-*	-	*-*	-	*-*	-	*-*	1	*1.43*	-	*-*
Landslide	15	*21.43*	2	*2.86*	-	*-*	-	*-*	12	*17.14*	1	*1.43*	-	*-*
Rock fall	1	*1.43*	-	*-*	-	*-*	-	*-*	1	*1.43*	-	*-*	-	*-*
Road collapse	1	*1.43*	-	*-*	-	*-*	-	*-*	1	*1.43*	-	*-*	-	*-*
Windstorm	9	*15.71*	-	*-*	-	*-*	-	*-*	6	*8.57*	3	*4.29*	-	*-*
Lightning	10	*14.29*	-	*-*	4	*5.71*	-	*-*	5	*7.14*	-	*-*	1	*1.43*
Storm surge	5	*7.14*	-	*-*	-	*-*	-	*-*	3	*4.29*	1	*1.43*	1	*1.43*
Others (hail, thunderstorm)	-	*-*	-	*-*	-	*-*	-	*-*	-	*-*	-	*-*	-	*-*
**Injured**														
**Type of Phenomenon**	**Total Injured**		**0****–****10 Y**		**11****–****18 Y**		**19****–****25 Y**		**26****–****65 Y**		**>65 Y**		**Unknown**	
	**#**	***%***	**#**	***%***	**#**	***%***	**#**	***%***	**#**	***%***	**#**	***%***	**#**	***%***
Flood	194	*34.28*	5	*0.88*	7	*1.24*	10	*1.77*	39	*6.89*	13	*2.30*	120	*21.20*
Urban flooding	12	*2.12*	-	*-*	-	*-*	1	*0.18*	2	*0.35*	4	*0.71*	5	*0.88*
Landslide	178	*31.63*	3	*0.53*	1	*0.18*	9	*1.59*	44	*7.77*	8	*1.41*	113	*19.96*
Rock fall	5	*0.88*	-	*-*	1	*0.18*	-	*-*	1	*0.18*	-	*-*	3	*0.53*
Road collapse	16	*2.83*	-	*-*	-	*-*	1	*0.18*	9	*1.59*	1	*0.18*	5	*0.88*
Windstorm	93	*16.43*	4	*0.71*	-	*-*	10	*1.77*	26	*4.59*	5	*0.88*	48	*8.48*
Lightning	32	*5.65*	3	*0.53*	6	*1.06*	2	*0.35*	11	*1.94*	-	*-*	10	*1.77*
Storm surge	18	*3.18*	5	*0.88*	1	*0.18*	1	*0.18*	2	*0.35*	-	*-*	9	*1.59*
Others (hail, thunderstorm)	18	*3.00*	-	*-*	-	*-*	-	*-*	3	*0.53*	2	*0.35*	13	*2.30*
**Involved**														
**Type of Phenomenon**	**Total Involved**		**0****–****10 Y**		**11****–****18 Y**		**19****–****25 Y**		**26****–****65 Y**		**>65 Y**		**Unknown**	
	**#**	***%***	**#**	***%***	**#**	***%***	**#**	***%***	**#**	***%***	**#**	***%***	**#**	***%***
Flood	2084	*31.33*	26	*0.39*	76	*1.14*	14	*0.21*	100	*1.50*	35	*0.53*	1833	*27.56*
Urban flooding	1961	*29.48*	249	*3.74*	123	*1.85*	5	*0.08*	39	*0.59*	47	*0.71*	1498	*22.52*
Landslide	1949	*29.30*	46	*0.69*	58	*0.87*	7	*0.11*	58	*0.87*	11	*0.17*	1769	*26.59*
Rock fall	49	*0.74*	-	*-*	-	*-*	1	*0.02*	2	*0.03*	1	*0.02*	45	*0.68*
Road collapse	95	*1.43*	4	*0.06*	10	*0.15*	4	*0.06*	2	*0.03*	-	*-*	75	*1.13*
Windstorm	259	*3.86*	7	*0.11*	4	*0.06*	-	*-*	32	*0.48*	5	*0.08*	211	*3.17*
Lightning	44	*0.66*	-	*-*	-	*-*	1	*0.02*	-	*-*	-	*-*	43	*0.65*
Storm surge	92	*1.38*	2	*0.03*	3	*0.05*	1	*0.02*	13	*0.20*	8	*0.12*	65	*0.98*
Others (hail, thunderstorm)	121	*1.82*	4	*0.06*	-	*-*	1	*0.02*	-	*-*	2	*0.03*	114	*1.71*

Percentages in italics.

**Table A2:** Place, condition, activity and dynamic.

Place	Total People		Fatalities		Injured		Involved	
	#	*%*	#	*%*	#	*%*	#	*%*
Bungalow	70	*0.96*	11	*16.18*	46	*8.13*	13	*0.32*
Hotel	162	*2.22*	-	*-*	21	*3.71*	141	*2.12*
Public/private building	3128	*42.92*	7	*10.29*	51	*9.01*	3070	*46.14*
School	160	*2.20*	-	*-*	-	*-*	160	*2.40*
Shop	20	*0.27*	-	*-*	-	*-*	20	*0.30*
Road	2520	*34.58*	21	*30.88*	183	*32.33*	2308	*34.69*
Countryside	150	*2.06*	12	*17.65*	17	*3.00*	129	*1.82*
Railway	527	*7.23*	5	*7.35*	83	*14.66*	439	*6.60*
Riverbed/riverside	49	*0.67*	5	*7.35*	4	*0.71*	40	*0.60*
Beach	49	*0.67*	3	*4.41*	21	*3.71*	25	*0.38*
Campsite/tent	136	*1.87*	2	*2.94*	9	*1.59*	125	*1.88*
Port	7	*0.10*	1	*1.47*	2	*0.35*	4	*0.06*
Recreation area	17	*0.23*	1	*1.47*	12	*2,12*	4	*0.06*
Underpass	137	*1.88*	-	*-*	-	*-*	137	*2.06*
Not reported	156	*2.14*	-	*-*	117	*20.67*	39	*0.59*
**Condition**								
Standing	3119	*42.80*	25	*36.76*	138	*24.38*	2956	*44.42*
By car	1892	*25.96*	14	*20.59*	99	*17.49*	1779	*26.74*
Laying	685	*9.40*	12	*17.65*	55	*9.72*	618	*9.29*
By train	626	*8.59*	4	*5.88*	83	*14.66*	539	*8.10*
By motorcycle	3	*0.04*	3	*4.41*	-	*-*	-	*-*
By van	98	*1.34*	3	*4.41*	9	*1.59*	86	*1.29*
By boat	11	*0.15*	1	*1.47*	-	*-*	10	*0.15*
By caravan	8	*0.11*	1	*1.47*	1	*0.18*	6	*0.09*
By tractor	14	*0.19*	1	*1.47*	4	*0.71*	9	*0.14*
By truck	82	*1.13*	1	*1.47*	18	*3.18*	63	*0.95*
By bus	390	*5.35*	-	*-*	26	*4.59*	364	*5.47*
By bicycle	1	*0.01*	-	*-*	1	*0.18*	-	*-*
Not reported	359	*4.93*	3	*4.41*	132	*23.32*	224	*3.37*
**Activity**								
Working	707	*9.70*	16	*23.53*	59	*10.42*	632	*9.50*
Travelling	2848	*39.08*	15	*22.06*	202	*35.69*	2631	*39.54*
Sleeping	882	*12.10*	13	*19.12*	54	*9.54*	815	*12.25*
Recreational activities	208	*2.85*	5	*7.35*	52	*9.19*	151	*2.27*
Rescuing someone	20	*0.27*	3	*4.41*	7	*1.24*	10	*0.15*
Fishing	2	*0.03*	1	*1.47*	1	*0.18*	-	*-*
Going at home	54	*0.74*	2	*2.94*	6	*1.06*	46	*0.69*
Going to work	7	*0.10*	2	*2.94*	3	*0.53*	2	*0.03*
Housework	2	*0.03*	1	*1.47*	0	*0.00*	1	*0.02*
Walking	64	*0.88*	-	*-*	10	*1.77*	54	*0.81*
Not reported	2494	*34.22*	10	*14.71*	172	*30.39*	2312	*34.75*
**Dynamic**								
Dragged by water/mud	143	*1.96*	29	*42.65*	17	*3.00*	97	*1.46*
Hit by lightning	46	*0.63*	9	*13.24*	27	*4.77*	10	*0.15*
Blocked in a flooded room	393	*5.39*	4	*5.88*	16	*2.83*	373	*5.61*
Caught in a derailment	361	*4.95*	4	*5.88*	71	*12.54*	286	*4.30*
Hit by landslide	484	*6.64*	4	*5.88*	73	*12.90*	407	*6.12*
Hurled by wind	55	*0.75*	4	*5.88*	18	*3.18*	33	*0.50*
Hit by objects broken by lightning	60	*0.82*	1	*1.47*	14	*2.47*	45	*0.68*
Buried	9	*0.12*	3	*4.41*	6	*1.06*	0	*0.00*
Fallen into the river	10	*0.14*	2	*2.94*	2	*0.35*	6	*0.09*
Hit by object hurled by wind	231	*3.17*	2	*2.94*	63	*11.13*	166	*2.49*
Swept away by sea waves	26	*0.36*	2	*2.94*	10	*1.77*	14	*0.21*
Caught in a road/bridge collapse	142	*1.95*	1	*1.47*	24	*4.24*	117	*1.76*
Caught in building collapse	372	*5.10*	1	*1.47*	5	*0.88*	366	*5.50*
Fallen down a slope	4	*0.05*	1	*1.47*	2	*0.35*	1	*0.02*
Surrounded by water/ mud	4224	*57.96*	1	*1.47*	63	*11.13*	4160	*62.52*
Hit by hail	25	*0.34*	-	*-*	16	*2.83*	9	*0.14*
Blocked by landslide debris	460	*6.31*	-	*-*	13	*2.30*	447	*6.72*
Blocked by object hurled by wind	79	*1.08*	-	*-*	1	*0.18*	78	*1.17*
Not reported	164	*2.25*	-	*-*	125	*22.08*	39	*0.59*

Percentages in italics.

**Table A3:** Gender, causes of death and type of injured.

**Cause of Death**	**Total**		**Males**		**Females**		**Unknown**	
	**#**	***%***	**#**	***%***	**#**	***%***	**#**	***%***
Drowning	29	*42.65*	21	*30.88*	8	*11.76*	-	*-*
Poly-trauma	16	*23.53*	14	*20.59*	2	*2.94*	-	*-*
Electrocuted	10	*14.71*	9	*13.24*	1	*1.47*	-	*-*
Poly-trauma and Suffocation	7	*10.29*	7	*10.29*	-	*-*	-	*-*
Heart attack	3	*4.41*	2	*2.94*	1	*1.47*	-	*-*
Collapse	1	*1.47*	1	*1.47*	-	*-*	-	*-*
Hypothermia	1	*1.47*	0	*0.00*	1	*1.47*	-	*-*
Suffocation	1	*1.47*	1	*1.47*	-	*-*	-	*-*
Unknown	-	*-*	-	*-*	-	*-*	-	*-*
**Type of Injury**	**Total**		**Males**		**Females**		**Unknown**	
Contusion and abrasion	108	*19.08*	43	*7.60*	13	*2.30*	52	*9.19*
Shock	58	*10.25*	17	*3.00*	9	*1.59*	32	*5.65*
Contusion	56	*9.89*	18	*3.18*	4	*0.71*	34	*6.01*
Contusion and shock	49	*8.66*	6	*1.06*	3	*0.53*	40	*7.07*
Abrasion	28	*4.95*	10	*1.77*	4	*0.71*	14	*2.47*
Abrasion and shock	28	*4.95*	11	*1.94*	15	*2.65*	2	*0.35*
Burns and shock	15	*2.65*	11	*1.94*	4	*0.71*	-	*-*
Poly fractures	12	*2.12*	10	*1.77*	1	*0.18*	1	*0.18*
Cranial trauma	11	*1.94*	9	*1.59*	2	*0.35*	-	*-*
Wounded limbs and cranial trauma	12	*2.12*	11	*1.94*	-	*-*	1	*0.18*
Fractured limbs	6	*1.06*	3	*0.53*	3	*0.53*	-	*-*
Fainting	4	*0.71*	2	*0.35*	2	*0.35*	-	*-*
Thoracic trauma	3	*0.53*	1	*0.18*	2	*0.35*	-	*-*
Near drowning	3	*0.53*	2	*0.35*	1	*0.18*	-	*-*
Frozen	1	*0.18*	1	*0.18*	-	*-*	-	*-*
Unknown	172	*30.39*	1	*0.18*	-	*-*	-	*-*

Percentages in italics.

**Table A4:** Types of phenomena and hazardous behaviors.

Phenomenon	Hazardous Behavior	Fatalities	Injured	Involved
		#	#	#
Flood	Fording rivers	2		23
	Staying on river banks	2		
	Trying to save vehicles			3
	Enter/stay in floooded underpass			121
Landslide	Refuse evacuation			10
Windstorm	Refuse warnings		1	2
Lightning	Under a tree during lightning	3	2	4
Storm surge	Check damage during the event	1		1
	Trying to save belongings	1		5
**Total**		**9**	**3**	**169**
